# *N*′-(4-Chloro­benzyl­idene)-2-hydroxy­benzohydrazide

**DOI:** 10.1107/S160053680803972X

**Published:** 2008-12-10

**Authors:** Shu-Ping Zhang, Rui Qiao, Si-Chang Shao

**Affiliations:** a Department of Chemistry, Fuyang Normal College, Fuyang Anhui 236041, People’s Republic of China

## Abstract

The title mol­ecule, C_14_H_11_ClN_2_O_2_, adopts a *trans* configuration with respect to the C=N double bond. An intra­molecular N—H⋯O hydrogen bond contributes to mol­ecular conformation and the two benzene rings form a dihedral angle of 17.9 (8)°. In the crystal structure, inter­molecular O—H⋯O hydrogen bonds link the mol­ecules into chains running along [10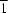].

## Related literature

For general background to hydrazones and Schiff bases and their potential pharmacological and anti­tumor properties, see: Karthikeyan *et al.* (2006 ▶); Khattab (2005 ▶); Kucukguzel *et al.* (2006 ▶); Okabe *et al.* (1993 ▶).
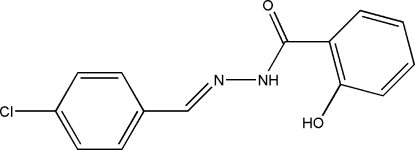


## Experimental

### 

#### Crystal data

C_14_H_11_ClN_2_O_2_
*M* *_r_* = 274.70
Monoclinic, 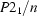

*a* = 4.8557 (6) Å
*b* = 24.588 (3) Å
*c* = 11.0903 (13) Å
β = 99.710 (2)°
*V* = 1305.1 (3) Å^3^
*Z* = 4
Mo *K*α radiation
μ = 0.29 mm^−1^
*T* = 298 (2) K
0.10 × 0.10 × 0.08 mm

#### Data collection

Bruker SMART CCD area-detector diffractometer
Absorption correction: multi-scan (*SADABS*; Sheldrick, 1996 ▶) *T*_min_ = 0.972, *T*_max_ = 0.977
11227 measured reflections
3126 independent reflections
2402 reflections with *I* > 2σ(*I*)
*R*_int_ = 0.027

#### Refinement

*R*[*F* ^2^ > 2σ(*F* ^2^)] = 0.067
*wR*(*F* ^2^) = 0.169
*S* = 1.12
3126 reflections
180 parameters
1 restraint
H atoms treated by a mixture of independent and constrained refinement
Δρ_max_ = 0.38 e Å^−3^
Δρ_min_ = −0.22 e Å^−3^

### 

Data collection: *SMART* (Siemens, 1996 ▶); cell refinement: *SAINT* (Siemens, 1996 ▶); data reduction: *SAINT*; program(s) used to solve structure: *SHELXS97* (Sheldrick, 2008 ▶); program(s) used to refine structure: *SHELXL97* (Sheldrick, 2008 ▶); molecular graphics: *SHELXTL* (Sheldrick, 2008 ▶); software used to prepare material for publication: *SHELXTL*.

## Supplementary Material

Crystal structure: contains datablocks global, I. DOI: 10.1107/S160053680803972X/cv2476sup1.cif

Structure factors: contains datablocks I. DOI: 10.1107/S160053680803972X/cv2476Isup2.hkl

Additional supplementary materials:  crystallographic information; 3D view; checkCIF report

## Figures and Tables

**Table 1:** Hydrogen-bond geometry (Å, °)

*D*—H⋯*A*	*D*—H	H⋯*A*	*D*⋯*A*	*D*—H⋯*A*
N1—H2⋯O1	0.80 (3)	2.01 (3)	2.624 (2)	134 (2)
O1—H1⋯O2^i^	0.782 (18)	1.90 (2)	2.647 (2)	159 (3)

Symmetry code: (i) 
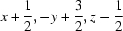
.

**Fig. 1.**
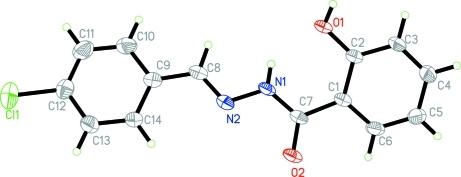
The molecular structure of (I), showing 30% probability displacement ellipsoids and the atom-numbering scheme.

## supplementary crystallographic information

### Comment

Hydrazones and Schiff bases have attracted much attention for their excellent biological properties, especially for their potential pharmacological and antitumor properties (Kucukguzel *et al.*, 2006; Khattab, 2005; Karthikeyan *et al.*, 2006; Okabe *et al.*, 1993). We are interested in this fields. As a part of ongoing study, we report herein the crystal structure of the title compound, (I).

The molecular structure of (I) (Fig. 1) displays a *trans* configuration about the C=N bond. Intramolecular N—H···O hydrogen bond (Table 1) contributes to molecular conformation - the dihedral angle between the two benzene rings is 17.9 (8)°. In the crystal, the molecules are linked into chains by intermolecular O—H···O hydrongen bonds (Table 1).

### Experimental

Equivalent amounts of 2-Hydroxybenzohydrazide and 3-chlorobenzohydrazide were reacted in ethanol (10 mL) for 1 h. After allowing the resulting solution to stand in air for 10 d colourless block-shaped crystals were formed on slow evaporation of the solvent.

### Refinement

C-bound H atoms were placed in calculated positions (C—H = 0.93 Å) and constrained to ride on their parent atom, with U_iso_(H) = 1.2U_eq_(C). The remaining H atoms were located in a difference map and refined isotropically.

### Crystal data

**Table:**

C_14_H_11_ClN_2_O_2_	*F*(000) = 568
*M**_r_* = 274.70	*D*_x_ = 1.398 Mg m^−^^3^
Monoclinic, *P*2_1_/*n*	Mo *K*α radiation, λ = 0.71073 Å
*a* = 4.8557 (6) Å	Cell parameters from 986 reflections
*b* = 24.588 (3) Å	θ = 2.1–28.2°
*c* = 11.0903 (13) Å	µ = 0.29 mm^−^^1^
β = 99.710 (2)°	*T* = 298 K
*V* = 1305.1 (3) Å^3^	Block, colourless
*Z* = 4	0.10 × 0.10 × 0.08 mm

### Data collection

**Table:**

Bruker SMART CCD area-detector diffractometer	3126 independent reflections
Radiation source: fine-focus sealed tube	2402 reflections with *I* > 2σ(*I*)
graphite	*R*_int_ = 0.027
φ and ω scans	θ_max_ = 28.2°, θ_min_ = 1.7°
Absorption correction: multi-scan (*SADABS*; Sheldrick, 1996)	*h* = −6→6
*T*_min_ = 0.972, *T*_max_ = 0.977	*k* = −32→31
11227 measured reflections	*l* = −14→13

### Refinement

**Table:**

Refinement on *F*^2^	Primary atom site location: structure-invariant direct methods
Least-squares matrix: full	Secondary atom site location: difference Fourier map
*R*[*F*^2^ > 2σ(*F*^2^)] = 0.067	Hydrogen site location: inferred from neighbouring sites
*wR*(*F*^2^) = 0.169	H atoms treated by a mixture of independent and constrained refinement
*S* = 1.12	*w* = 1/[σ^2^(*F*_o_^2^) + (0.0705*P*)^2^ + 0.4971*P*] where *P* = (*F*_o_^2^ + 2*F*_c_^2^)/3
3126 reflections	(Δ/σ)_max_ = 0.072
180 parameters	Δρ_max_ = 0.38 e Å^−^^3^
1 restraint	Δρ_min_ = −0.22 e Å^−^^3^

### Special details

**Table:**

Geometry. All e.s.d.'s (except the e.s.d. in the dihedral angle between two l.s. planes) are estimated using the full covariance matrix. The cell e.s.d.'s are taken into account individually in the estimation of e.s.d.'s in distances, angles and torsion angles; correlations between e.s.d.'s in cell parameters are only used when they are defined by crystal symmetry. An approximate (isotropic) treatment of cell e.s.d.'s is used for estimating e.s.d.'s involving l.s. planes.
Refinement. Refinement of *F*^2^ against ALL reflections. The weighted *R*-factor *wR* and goodness of fit *S* are based on *F*^2^, conventional *R*-factors *R* are based on *F*, with *F* set to zero for negative *F*^2^. The threshold expression of *F*^2^ > σ(*F*^2^) is used only for calculating *R*-factors(gt) *etc*. and is not relevant to the choice of reflections for refinement. *R*-factors based on *F*^2^ are statistically about twice as large as those based on *F*, and *R*- factors based on ALL data will be even larger.

### Fractional atomic coordinates and isotropic or equivalent isotropic displacement parameters (Å^2^)

**Table:**

	*x*	*y*	*z*	*U*_iso_*/*U*_eq_	
Cl1	−0.39445 (17)	1.00945 (3)	0.73436 (9)	0.0851 (3)	
O1	0.9734 (4)	0.73796 (8)	0.50406 (15)	0.0610 (5)	
N1	0.6996 (4)	0.76768 (8)	0.67797 (18)	0.0476 (5)	
O2	0.8154 (4)	0.72610 (8)	0.85960 (14)	0.0618 (5)	
N2	0.5245 (4)	0.80391 (8)	0.72149 (16)	0.0465 (5)	
C7	0.8405 (4)	0.73021 (9)	0.75183 (18)	0.0442 (5)	
C1	1.0293 (4)	0.69349 (9)	0.69718 (18)	0.0411 (5)	
C8	0.3878 (5)	0.83421 (10)	0.6399 (2)	0.0507 (6)	
H8	0.4112	0.8294	0.5591	0.061*	
C3	1.2815 (5)	0.66229 (11)	0.5401 (2)	0.0531 (6)	
H3	1.3252	0.6657	0.4619	0.064*	
C2	1.0942 (4)	0.69832 (9)	0.57896 (18)	0.0423 (5)	
C4	1.4019 (5)	0.62186 (11)	0.6157 (2)	0.0588 (6)	
H4	1.5299	0.5984	0.5892	0.071*	
C9	0.1965 (5)	0.87608 (10)	0.6674 (2)	0.0485 (5)	
C6	1.1533 (5)	0.65119 (11)	0.7705 (2)	0.0562 (6)	
H6	1.1112	0.6471	0.8487	0.067*	
C13	−0.0225 (6)	0.93008 (11)	0.8045 (3)	0.0648 (7)	
H13	−0.0453	0.9389	0.8838	0.078*	
C14	0.1588 (6)	0.88896 (11)	0.7845 (2)	0.0571 (6)	
H14	0.2561	0.8698	0.8506	0.069*	
C12	−0.1684 (5)	0.95778 (10)	0.7069 (3)	0.0579 (6)	
C5	1.3350 (6)	0.61556 (12)	0.7309 (2)	0.0623 (7)	
H5	1.4127	0.5873	0.7812	0.075*	
C10	0.0436 (6)	0.90483 (13)	0.5712 (3)	0.0713 (8)	
H10	0.0643	0.8963	0.4915	0.086*	
C11	−0.1375 (6)	0.94547 (13)	0.5903 (3)	0.0763 (9)	
H11	−0.2377	0.9644	0.5245	0.092*	
H1	1.053 (6)	0.7435 (12)	0.449 (2)	0.081 (10)*	
H2	0.708 (5)	0.7692 (10)	0.607 (2)	0.051 (7)*	

### Atomic displacement parameters (Å^2^)

**Table:**

	*U*^11^	*U*^22^	*U*^33^	*U*^12^	*U*^13^	*U*^23^
Cl1	0.0716 (5)	0.0602 (5)	0.1249 (8)	0.0104 (3)	0.0203 (5)	0.0032 (4)
O1	0.0751 (11)	0.0777 (13)	0.0384 (9)	0.0191 (9)	0.0334 (8)	0.0132 (8)
N1	0.0595 (11)	0.0569 (12)	0.0316 (9)	0.0026 (9)	0.0223 (8)	−0.0021 (8)
O2	0.0829 (12)	0.0735 (12)	0.0359 (8)	0.0106 (9)	0.0301 (8)	0.0019 (8)
N2	0.0528 (10)	0.0519 (11)	0.0384 (10)	−0.0043 (8)	0.0186 (8)	−0.0053 (8)
C7	0.0513 (12)	0.0507 (13)	0.0346 (10)	−0.0096 (10)	0.0191 (9)	−0.0043 (9)
C1	0.0452 (11)	0.0483 (12)	0.0321 (10)	−0.0081 (9)	0.0136 (8)	−0.0037 (9)
C8	0.0585 (13)	0.0622 (15)	0.0342 (11)	−0.0036 (11)	0.0154 (10)	−0.0046 (10)
C3	0.0599 (13)	0.0668 (16)	0.0365 (11)	0.0045 (11)	0.0192 (10)	−0.0054 (11)
C2	0.0459 (11)	0.0513 (13)	0.0320 (10)	−0.0042 (9)	0.0133 (8)	−0.0009 (9)
C4	0.0632 (15)	0.0669 (16)	0.0471 (13)	0.0129 (12)	0.0117 (11)	−0.0114 (12)
C9	0.0514 (12)	0.0545 (14)	0.0401 (11)	−0.0073 (10)	0.0094 (9)	−0.0007 (10)
C6	0.0744 (16)	0.0621 (15)	0.0352 (11)	0.0040 (12)	0.0182 (11)	0.0028 (11)
C13	0.0829 (18)	0.0613 (16)	0.0554 (15)	0.0062 (14)	0.0266 (13)	0.0019 (13)
C14	0.0732 (16)	0.0579 (15)	0.0432 (13)	0.0118 (12)	0.0182 (11)	0.0090 (11)
C12	0.0519 (13)	0.0492 (14)	0.0732 (17)	−0.0039 (11)	0.0120 (12)	0.0066 (12)
C5	0.0808 (18)	0.0614 (16)	0.0441 (13)	0.0150 (13)	0.0084 (12)	0.0032 (12)
C10	0.0768 (18)	0.090 (2)	0.0453 (14)	0.0106 (16)	0.0059 (12)	0.0040 (14)
C11	0.0708 (18)	0.090 (2)	0.0633 (18)	0.0143 (16)	−0.0019 (14)	0.0168 (16)

### Geometric parameters (Å, °)

**Table:**

Cl1—C12	1.739 (3)	C4—C5	1.379 (3)
O1—C2	1.349 (3)	C4—H4	0.9300
O1—H1	0.782 (18)	C9—C14	1.379 (3)
N1—C7	1.341 (3)	C9—C10	1.386 (4)
N1—N2	1.374 (3)	C6—C5	1.367 (3)
N1—H2	0.80 (3)	C6—H6	0.9300
O2—C7	1.226 (2)	C13—C12	1.371 (4)
N2—C8	1.269 (3)	C13—C14	1.383 (4)
C7—C1	1.487 (3)	C13—H13	0.9300
C1—C6	1.393 (3)	C14—H14	0.9300
C1—C2	1.404 (3)	C12—C11	1.360 (4)
C8—C9	1.453 (3)	C5—H5	0.9300
C8—H8	0.9300	C10—C11	1.371 (4)
C3—C4	1.367 (3)	C10—H10	0.9300
C3—C2	1.389 (3)	C11—H11	0.9300
C3—H3	0.9300		
			
C2—O1—H1	112 (2)	C14—C9—C8	123.4 (2)
C7—N1—N2	120.86 (18)	C10—C9—C8	118.6 (2)
C7—N1—H2	122.0 (18)	C5—C6—C1	122.0 (2)
N2—N1—H2	117.1 (18)	C5—C6—H6	119.0
C8—N2—N1	114.23 (18)	C1—C6—H6	119.0
O2—C7—N1	121.9 (2)	C12—C13—C14	119.7 (2)
O2—C7—C1	121.1 (2)	C12—C13—H13	120.2
N1—C7—C1	117.02 (17)	C14—C13—H13	120.2
C6—C1—C2	117.7 (2)	C9—C14—C13	120.5 (2)
C6—C1—C7	116.82 (18)	C9—C14—H14	119.7
C2—C1—C7	125.5 (2)	C13—C14—H14	119.7
N2—C8—C9	122.9 (2)	C11—C12—C13	121.0 (3)
N2—C8—H8	118.6	C11—C12—Cl1	120.2 (2)
C9—C8—H8	118.6	C13—C12—Cl1	118.8 (2)
C4—C3—C2	120.5 (2)	C6—C5—C4	119.4 (2)
C4—C3—H3	119.7	C6—C5—H5	120.3
C2—C3—H3	119.7	C4—C5—H5	120.3
O1—C2—C3	120.60 (18)	C11—C10—C9	121.8 (3)
O1—C2—C1	119.55 (19)	C11—C10—H10	119.1
C3—C2—C1	119.9 (2)	C9—C10—H10	119.1
C3—C4—C5	120.4 (2)	C12—C11—C10	119.1 (3)
C3—C4—H4	119.8	C12—C11—H11	120.5
C5—C4—H4	119.8	C10—C11—H11	120.5
C14—C9—C10	117.9 (2)		
			
C7—N1—N2—C8	−174.7 (2)	N2—C8—C9—C10	−176.0 (2)
N2—N1—C7—O2	1.3 (3)	C2—C1—C6—C5	0.7 (4)
N2—N1—C7—C1	−178.79 (18)	C7—C1—C6—C5	−178.7 (2)
O2—C7—C1—C6	6.2 (3)	C10—C9—C14—C13	−1.2 (4)
N1—C7—C1—C6	−173.7 (2)	C8—C9—C14—C13	178.6 (2)
O2—C7—C1—C2	−173.2 (2)	C12—C13—C14—C9	0.8 (4)
N1—C7—C1—C2	6.9 (3)	C14—C13—C12—C11	−0.1 (4)
N1—N2—C8—C9	−178.5 (2)	C14—C13—C12—Cl1	−179.8 (2)
C4—C3—C2—O1	−179.4 (2)	C1—C6—C5—C4	1.0 (4)
C4—C3—C2—C1	0.5 (4)	C3—C4—C5—C6	−2.0 (4)
C6—C1—C2—O1	178.4 (2)	C14—C9—C10—C11	0.9 (4)
C7—C1—C2—O1	−2.2 (3)	C8—C9—C10—C11	−178.9 (3)
C6—C1—C2—C3	−1.5 (3)	C13—C12—C11—C10	−0.2 (4)
C7—C1—C2—C3	177.9 (2)	Cl1—C12—C11—C10	179.5 (2)
C2—C3—C4—C5	1.3 (4)	C9—C10—C11—C12	−0.2 (5)
N2—C8—C9—C14	4.1 (4)		

### Hydrogen-bond geometry (Å, °)

**Table:**

*D*—H···*A*	*D*—H	H···*A*	*D*···*A*	*D*—H···*A*
N1—H2···O1	0.80 (3)	2.01 (3)	2.624 (2)	134 (2)
O1—H1···O2^i^	0.78 (2)	1.90 (2)	2.647 (2)	159 (3)

Symmetry codes: (i) *x*+1/2, −*y*+3/2, *z*−1/2.
